# (2*E*)-3-(4-Bromo­phen­yl)-1-(4,4′′-difluoro-5′-meth­oxy-1,1′:3′,1′′-terphenyl-4′-yl)prop-2-en-1-one

**DOI:** 10.1107/S1600536812046831

**Published:** 2012-11-24

**Authors:** Seranthimata Samshuddin, Badiadka Narayana, Hemmige S. Yathirajan, Thomas Gerber, Eric Hosten, Richard Betz

**Affiliations:** a Mangalore University, Department of Studies in Chemistry, Mangalagangotri 574 199, India; b University of Mysore, Department of Studies in Chemistry, Manasagangotri, Mysore 570 006, India; c Nelson Mandela Metropolitan University, Summerstrand Campus, Department of Chemistry, University Way, Summerstrand, PO Box 77000, Port Elizabeth, 6031, South Africa

## Abstract

In the title compound, C_28_H_19_BrF_2_O_2_, the C=C double bond is *E*-configured. In the crystal, C—H⋯O and C—H⋯F contacts connect mol­ecules into planes perpendicular to the *c* axis. The shortest centroid–centroid distance between two aromatic systems is 3.6745 (12) Å between one of the *para*-fluoro­phenyl rings and its symmetry-generated equivalent.

## Related literature

For background to polysubstituted aromatics, see: Astrue (2002 ▶). For the pharmacological properties of terphenyls, see: Liu (2006 ▶). For the crystal structures of various terphenyl chalcones, see: Fun *et al.* (2012*a* ▶,*b* ▶,*c* ▶,*d* ▶,*e* ▶); Betz *et al.* (2011*a* ▶,*b* ▶,*c* ▶,*d* ▶,*e* ▶). For graph-set analysis of hydrogen bonds, see: Etter *et al.* (1990 ▶); Bernstein *et al.* (1995 ▶).
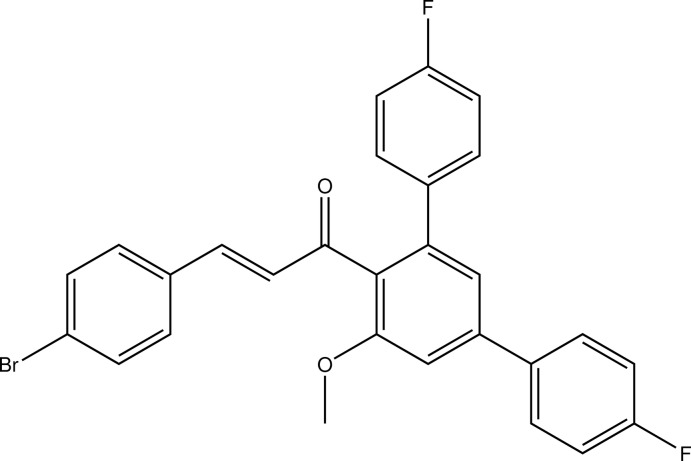


## Experimental

### 

#### Crystal data

C_28_H_19_BrF_2_O_2_
*M* *_r_* = 505.34
Triclinic, 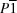

*a* = 6.9648 (2) Å
*b* = 11.3616 (3) Å
*c* = 14.7219 (4) Å
α = 95.983 (1)°
β = 92.601 (1)°
γ = 105.676 (1)°
*V* = 1112.23 (5) Å^3^
*Z* = 2
Mo *K*α radiation
μ = 1.89 mm^−1^
*T* = 200 K
0.39 × 0.18 × 0.06 mm

#### Data collection

Bruker APEXII CCD diffractometer
Absorption correction: multi-scan (*SADABS*; Bruker, 2008 ▶) *T*_min_ = 0.843, *T*_max_ = 1.000
20205 measured reflections
5513 independent reflections
4082 reflections with *I* > 2σ(*I*)
*R*_int_ = 0.021

#### Refinement

*R*[*F* ^2^ > 2σ(*F* ^2^)] = 0.035
*wR*(*F* ^2^) = 0.089
*S* = 1.03
5513 reflections
299 parameters
H-atom parameters constrained
Δρ_max_ = 0.52 e Å^−3^
Δρ_min_ = −0.59 e Å^−3^

### 

Data collection: *APEX2* (Bruker, 2010 ▶); cell refinement: *SAINT* (Bruker, 2010 ▶); data reduction: *SAINT*; program(s) used to solve structure: *SHELXS97* (Sheldrick, 2008 ▶); program(s) used to refine structure: *SHELXL97* (Sheldrick, 2008 ▶); molecular graphics: *ORTEP-3* (Farrugia, 2012 ▶) and *Mercury* (Macrae *et al.*, 2008 ▶); software used to prepare material for publication: *SHELXL97* and *PLATON* (Spek, 2009 ▶).

## Supplementary Material

Crystal structure: contains datablock(s) I, global. DOI: 10.1107/S1600536812046831/kj2214sup1.cif

Supplementary material file. DOI: 10.1107/S1600536812046831/kj2214Isup2.cdx

Structure factors: contains datablock(s) I. DOI: 10.1107/S1600536812046831/kj2214Isup3.hkl

Supplementary material file. DOI: 10.1107/S1600536812046831/kj2214Isup4.cml

Additional supplementary materials:  crystallographic information; 3D view; checkCIF report

## Figures and Tables

**Table 1:** Hydrogen-bond geometry (Å, °)

*D*—H⋯*A*	*D*—H	H⋯*A*	*D*⋯*A*	*D*—H⋯*A*
C4—H4*B*⋯F1^i^	0.98	2.51	3.428 (2)	156
C25—H25⋯O1^ii^	0.95	2.43	3.268 (2)	147

Symmetry codes: (i) 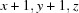; (ii) 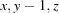.

**Fig. 1.**
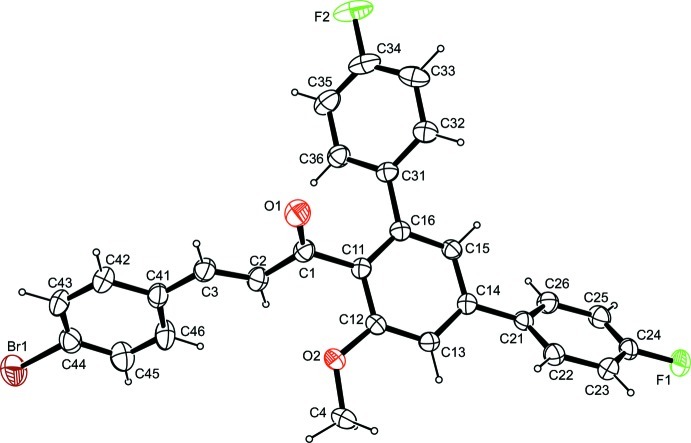
The molecular structure of the title compound, with atom labels and anisotropic displacement ellipsoids (drawn at 50% probability level).

**Fig. 2.**
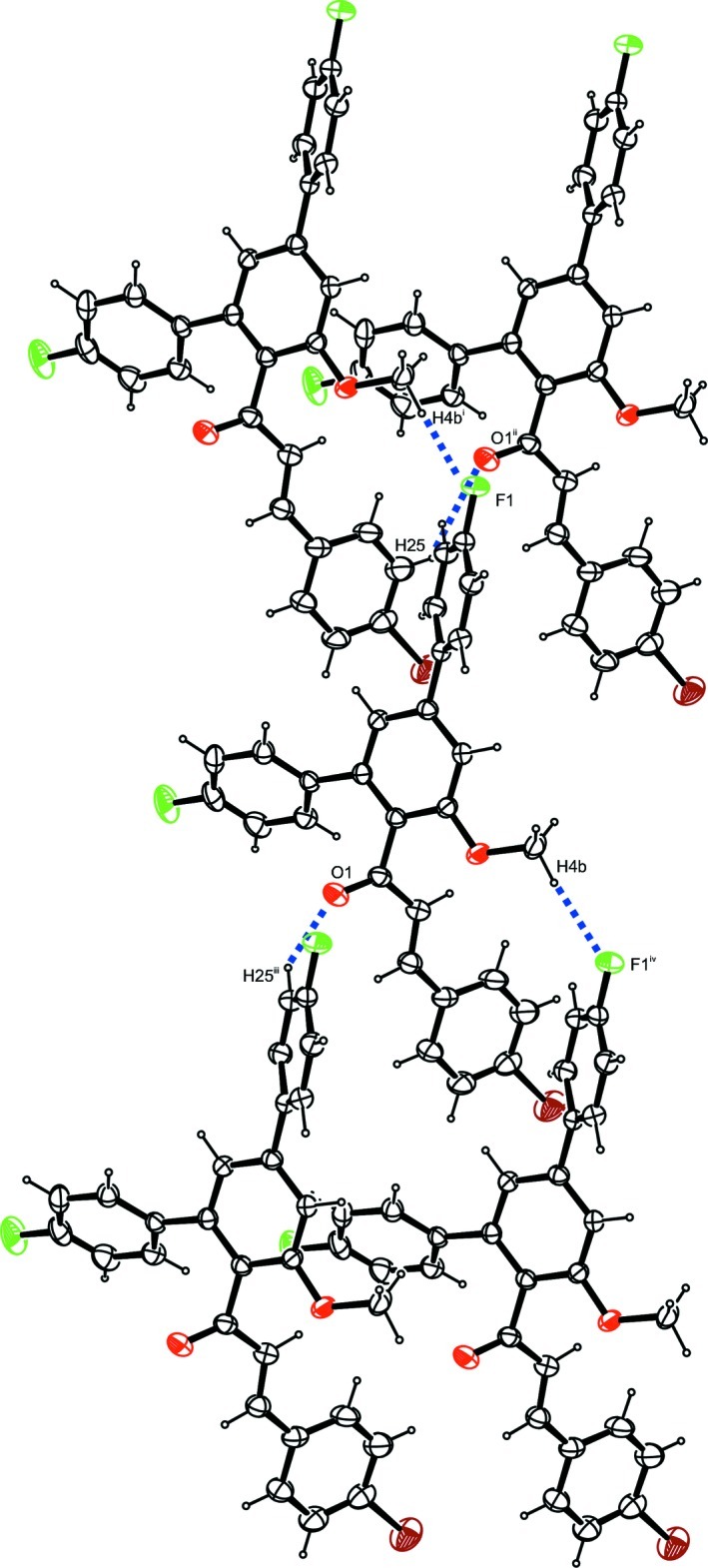
Intermolecular contacts, viewed approximately along [1 1 1]. Symmetry operators: ix - 1, y - 1, z; iix, y - 1, z; iiix, y + 1, z; ivx + 1, y + 1, z.

## supplementary crystallographic information

### Comment

The synthesis of polysubstituted aromatics has been and is a fascinating area in the field of organic chemistry (Astrue, 2002). In recent years, it has been reported that some terphenyls exhibit considerable biological activities such as anticoagulant, immunosuppressant, antithrombotic, neuroprotective, specific 5-lipoxygenase inhibitory and cytotoxic activities (Liu, 2006). Recently, the crystal structures of several terphenyl derivatives have been reported (Fun *et al.*, 2012*a*,*b*,*c*,*d*,*e*; Betz *et al.*, 2011*a*,*b*,*c*,*d*,*e*). In continuation of our ongoing interest in terphenyl derivatives, the title compound was prepared and its crystal structure determined.

The C=C double of the Michael system is (*E*)-configured. The least-squares planes defined by the carbon atoms of the *para*-fluoro phenyl rings of the terphenyl moiety and its central phenyl ring enclose angles of 41.74 (9)° and 46.49 (9)°, respectively (Fig. 1).

In the crystal structure, C–H···O as well as C–H···F contacts are present whose range falls by up to 0.2 Å below the sum of van-der-Waals radii of the atoms participating in them. While the C–H···O contacts are apparent between the ketonic oxygen atom and one of the *para*-fluorophenyl-bonded hydrogen atoms, the C–H···F contacts are supported by one of the hydrogen atoms of the methoxy substituent on the terphenyl's central phenyl group. In terms of graph-set analysis (Etter *et al.*, 1990; Bernstein *et al.*, 1995), the C–H···O contacts necessitate a *C*^1^_1_(10) descriptor on the unitary level and the C–H···F contacts necessitate a *C*^1^_1_(11) descriptor on the same level. These two antidromic chains connect the molecules to planes perpendicular to the crystallographic *c* axis. Details about metrical parameters of these contacts as well as information about their symmetry can be found in Table 1. The shortest intercentroid distance between two aromatic systems was found at 3.6745 (12) Å and is apparent between one of the *para*-fluoro phenyl moieties and its symmetry-generated equivalent (Fig. 2).

### Experimental

To a mixture of 1-(4,4''-difluoro-5'-methoxy-1,1':3',1''-terphenyl-4'-yl) ethanone (0.338 g, 0.001 mol) and 4-bromobenzaldehyde (0.185 g, 0.001 mol) in ethanol (20 ml), 10% sodium hydroxide solution (1 ml) was added and stirred at 278–283 K for 3 h. The precipitate formed was collected by filtration and dried (yield: 89%). Single crystals suitable for the X-ray diffraction study were grown from a DMF-ethanol mixture (*v*:*v* = 1:1) by slow evaporation at room temperature.

### Refinement

Carbon-bound H atoms were placed in calculated positions (C—H 0.95 Å for aromatic and vinylic carbon atoms) and were included in the refinement in the riding model approximation, with *U*(H) set to 1.2*U*_eq_(C). The H atoms of the methyl groups were allowed to rotate with a fixed angle around the C—C bond to best fit the experimental electron density (HFIX 137 in the *SHELX* program suite (Sheldrick, 2008)), with *U*(H) set to 1.5*U*_eq_(C).

### Crystal data

**Table:**

C_28_H_19_BrF_2_O_2_	*Z* = 2
*M**_r_* = 505.34	*F*(000) = 512
Triclinic, *P*1	*D*_x_ = 1.509 Mg m^−^^3^
Hall symbol: -P 1	Melting point: 465 K
*a* = 6.9648 (2) Å	Mo *K*α radiation, λ = 0.71073 Å
*b* = 11.3616 (3) Å	Cell parameters from 9095 reflections
*c* = 14.7219 (4) Å	θ = 2.8–28.1°
α = 95.983 (1)°	µ = 1.89 mm^−^^1^
β = 92.601 (1)°	*T* = 200 K
γ = 105.676 (1)°	Platelet, yellow
*V* = 1112.23 (5) Å^3^	0.39 × 0.18 × 0.06 mm

### Data collection

**Table:**

Bruker APEXII CCD diffractometer	5513 independent reflections
Radiation source: fine-focus sealed tube	4082 reflections with *I* > 2σ(*I*)
Graphite monochromator	*R*_int_ = 0.021
φ and ω scans	θ_max_ = 28.3°, θ_min_ = 2.2°
Absorption correction: multi-scan (*SADABS*; Bruker, 2008)	*h* = −9→8
*T*_min_ = 0.843, *T*_max_ = 1.000	*k* = −14→15
20205 measured reflections	*l* = −14→19

### Refinement

**Table:**

Refinement on *F*^2^	Primary atom site location: structure-invariant direct methods
Least-squares matrix: full	Secondary atom site location: difference Fourier map
*R*[*F*^2^ > 2σ(*F*^2^)] = 0.035	Hydrogen site location: inferred from neighbouring sites
*wR*(*F*^2^) = 0.089	H-atom parameters constrained
*S* = 1.03	*w* = 1/[σ^2^(*F*_o_^2^) + (0.0367*P*)^2^ + 0.5261*P*] where *P* = (*F*_o_^2^ + 2*F*_c_^2^)/3
5513 reflections	(Δ/σ)_max_ = 0.001
299 parameters	Δρ_max_ = 0.52 e Å^−^^3^
0 restraints	Δρ_min_ = −0.59 e Å^−^^3^

### Fractional atomic coordinates and isotropic or equivalent isotropic displacement parameters (Å^2^)

**Table:**

	*x*	*y*	*z*	*U*_iso_*/*U*_eq_	
Br1	1.47038 (4)	0.69865 (2)	0.964665 (19)	0.06360 (11)	
F1	0.17425 (18)	−0.70616 (9)	0.46554 (9)	0.0449 (3)	
F2	−0.2992 (2)	0.05818 (15)	0.95348 (10)	0.0660 (4)	
O1	0.2956 (2)	0.21292 (12)	0.70234 (10)	0.0411 (3)	
O2	0.68643 (19)	0.09212 (11)	0.60575 (9)	0.0333 (3)	
C1	0.4330 (3)	0.16804 (16)	0.71582 (12)	0.0283 (4)	
C2	0.6299 (3)	0.24098 (16)	0.75928 (12)	0.0311 (4)	
H2	0.7276	0.1995	0.7724	0.037*	
C3	0.6763 (3)	0.36236 (17)	0.78083 (13)	0.0333 (4)	
H3	0.5760	0.4015	0.7668	0.040*	
C4	0.8438 (3)	0.06260 (18)	0.55674 (13)	0.0354 (4)	
H4A	0.9021	0.0090	0.5905	0.053*	
H4B	0.9473	0.1386	0.5504	0.053*	
H4C	0.7895	0.0200	0.4959	0.053*	
C11	0.4061 (2)	0.03293 (15)	0.68905 (11)	0.0241 (3)	
C12	0.5376 (2)	−0.00254 (15)	0.62941 (11)	0.0254 (3)	
C13	0.5083 (2)	−0.12487 (15)	0.59477 (11)	0.0256 (3)	
H13	0.5959	−0.1470	0.5528	0.031*	
C14	0.3504 (3)	−0.21496 (15)	0.62163 (11)	0.0247 (3)	
C15	0.2252 (3)	−0.18096 (15)	0.68441 (11)	0.0256 (3)	
H15	0.1212	−0.2430	0.7050	0.031*	
C16	0.2490 (2)	−0.05819 (15)	0.71756 (11)	0.0242 (3)	
C21	0.3091 (2)	−0.34537 (15)	0.58143 (12)	0.0250 (3)	
C22	0.3101 (3)	−0.37450 (16)	0.48739 (12)	0.0294 (4)	
H22	0.3429	−0.3102	0.4495	0.035*	
C23	0.2639 (3)	−0.49584 (17)	0.44818 (13)	0.0318 (4)	
H23	0.2635	−0.5156	0.3838	0.038*	
C24	0.2190 (3)	−0.58645 (16)	0.50428 (14)	0.0318 (4)	
C25	0.2171 (3)	−0.56276 (17)	0.59744 (14)	0.0339 (4)	
H25	0.1863	−0.6279	0.6346	0.041*	
C26	0.2615 (3)	−0.44072 (16)	0.63582 (13)	0.0307 (4)	
H26	0.2594	−0.4220	0.7001	0.037*	
C31	0.1056 (3)	−0.02790 (16)	0.78209 (11)	0.0260 (3)	
C32	−0.0973 (3)	−0.08727 (18)	0.76598 (12)	0.0313 (4)	
H32	−0.1427	−0.1480	0.7145	0.038*	
C33	−0.2352 (3)	−0.0592 (2)	0.82402 (14)	0.0389 (5)	
H33	−0.3738	−0.1002	0.8130	0.047*	
C34	−0.1653 (3)	0.0291 (2)	0.89732 (14)	0.0422 (5)	
C35	0.0336 (3)	0.08800 (19)	0.91716 (14)	0.0422 (5)	
H35	0.0773	0.1478	0.9693	0.051*	
C36	0.1697 (3)	0.05844 (17)	0.85951 (12)	0.0327 (4)	
H36	0.3085	0.0975	0.8729	0.039*	
C41	0.8670 (3)	0.44118 (16)	0.82402 (13)	0.0337 (4)	
C42	0.8910 (3)	0.56700 (18)	0.84722 (15)	0.0420 (5)	
H42	0.7831	0.6004	0.8341	0.050*	
C43	1.0695 (3)	0.64393 (19)	0.88910 (15)	0.0463 (5)	
H43	1.0842	0.7294	0.9045	0.056*	
C44	1.2251 (3)	0.59503 (19)	0.90813 (13)	0.0406 (5)	
C45	1.2077 (4)	0.4715 (2)	0.88436 (16)	0.0467 (5)	
H45	1.3171	0.4390	0.8968	0.056*	
C46	1.0300 (3)	0.39593 (18)	0.84251 (15)	0.0432 (5)	
H46	1.0182	0.3111	0.8259	0.052*	

### Atomic displacement parameters (Å^2^)

**Table:**

	*U*^11^	*U*^22^	*U*^33^	*U*^12^	*U*^13^	*U*^23^
Br1	0.05179 (16)	0.05650 (17)	0.06461 (17)	−0.00644 (11)	−0.00125 (12)	−0.01426 (12)
F1	0.0442 (7)	0.0223 (5)	0.0631 (8)	0.0047 (5)	0.0065 (6)	−0.0069 (5)
F2	0.0665 (9)	0.0897 (11)	0.0614 (9)	0.0480 (8)	0.0353 (7)	0.0150 (8)
O1	0.0397 (8)	0.0304 (7)	0.0576 (9)	0.0162 (6)	0.0013 (7)	0.0093 (6)
O2	0.0317 (7)	0.0250 (6)	0.0404 (7)	0.0014 (5)	0.0131 (6)	0.0045 (5)
C1	0.0344 (10)	0.0236 (8)	0.0288 (8)	0.0095 (7)	0.0055 (7)	0.0064 (7)
C2	0.0360 (10)	0.0258 (9)	0.0319 (9)	0.0092 (7)	0.0016 (8)	0.0041 (7)
C3	0.0382 (10)	0.0275 (9)	0.0346 (9)	0.0094 (8)	0.0059 (8)	0.0027 (7)
C4	0.0262 (9)	0.0389 (10)	0.0394 (10)	0.0038 (8)	0.0102 (8)	0.0072 (8)
C11	0.0248 (8)	0.0231 (8)	0.0252 (8)	0.0081 (7)	−0.0006 (6)	0.0032 (6)
C12	0.0231 (8)	0.0250 (8)	0.0275 (8)	0.0046 (7)	0.0025 (7)	0.0055 (7)
C13	0.0240 (8)	0.0261 (8)	0.0275 (8)	0.0078 (7)	0.0041 (7)	0.0030 (7)
C14	0.0256 (8)	0.0235 (8)	0.0253 (8)	0.0076 (7)	−0.0005 (6)	0.0032 (6)
C15	0.0235 (8)	0.0249 (8)	0.0282 (8)	0.0054 (7)	0.0031 (6)	0.0048 (7)
C16	0.0233 (8)	0.0266 (8)	0.0236 (8)	0.0084 (7)	0.0002 (6)	0.0039 (6)
C21	0.0202 (8)	0.0231 (8)	0.0316 (9)	0.0062 (6)	0.0020 (7)	0.0024 (7)
C22	0.0285 (9)	0.0264 (9)	0.0326 (9)	0.0063 (7)	0.0033 (7)	0.0038 (7)
C23	0.0309 (9)	0.0303 (9)	0.0320 (9)	0.0073 (7)	0.0030 (7)	−0.0033 (7)
C24	0.0241 (9)	0.0219 (8)	0.0472 (11)	0.0054 (7)	0.0023 (8)	−0.0033 (8)
C25	0.0326 (10)	0.0250 (9)	0.0457 (11)	0.0078 (7)	0.0063 (8)	0.0099 (8)
C26	0.0322 (10)	0.0287 (9)	0.0320 (9)	0.0092 (7)	0.0040 (7)	0.0046 (7)
C31	0.0281 (9)	0.0265 (8)	0.0272 (8)	0.0121 (7)	0.0048 (7)	0.0077 (7)
C32	0.0299 (9)	0.0355 (10)	0.0313 (9)	0.0120 (8)	0.0039 (7)	0.0087 (8)
C33	0.0287 (10)	0.0533 (12)	0.0430 (11)	0.0187 (9)	0.0092 (8)	0.0211 (10)
C34	0.0500 (13)	0.0532 (13)	0.0381 (11)	0.0324 (11)	0.0210 (9)	0.0163 (9)
C35	0.0570 (14)	0.0402 (11)	0.0345 (10)	0.0211 (10)	0.0131 (9)	0.0026 (9)
C36	0.0364 (10)	0.0319 (9)	0.0306 (9)	0.0104 (8)	0.0050 (8)	0.0041 (7)
C41	0.0426 (11)	0.0245 (9)	0.0315 (9)	0.0051 (8)	0.0065 (8)	0.0021 (7)
C42	0.0468 (12)	0.0283 (10)	0.0494 (12)	0.0105 (9)	0.0081 (9)	−0.0047 (9)
C43	0.0547 (13)	0.0285 (10)	0.0483 (12)	0.0038 (9)	0.0129 (10)	−0.0122 (9)
C44	0.0436 (12)	0.0374 (11)	0.0325 (10)	−0.0005 (9)	0.0052 (8)	−0.0030 (8)
C45	0.0477 (13)	0.0373 (11)	0.0528 (13)	0.0079 (10)	−0.0022 (10)	0.0077 (10)
C46	0.0491 (12)	0.0233 (9)	0.0537 (13)	0.0063 (9)	−0.0039 (10)	0.0024 (9)

### Geometric parameters (Å, º)

**Table:**

Br1—C44	1.891 (2)	C22—H22	0.9500
F1—C24	1.3657 (19)	C23—C24	1.366 (3)
F2—C34	1.357 (2)	C23—H23	0.9500
O1—C1	1.217 (2)	C24—C25	1.371 (3)
O2—C12	1.364 (2)	C25—C26	1.388 (2)
O2—C4	1.433 (2)	C25—H25	0.9500
C1—C2	1.474 (3)	C26—H26	0.9500
C1—C11	1.503 (2)	C31—C32	1.389 (3)
C2—C3	1.329 (3)	C31—C36	1.395 (2)
C2—H2	0.9500	C32—C33	1.392 (3)
C3—C41	1.460 (3)	C32—H32	0.9500
C3—H3	0.9500	C33—C34	1.368 (3)
C4—H4A	0.9800	C33—H33	0.9500
C4—H4B	0.9800	C34—C35	1.369 (3)
C4—H4C	0.9800	C35—C36	1.385 (3)
C11—C16	1.402 (2)	C35—H35	0.9500
C11—C12	1.404 (2)	C36—H36	0.9500
C12—C13	1.387 (2)	C41—C46	1.394 (3)
C13—C14	1.389 (2)	C41—C42	1.397 (3)
C13—H13	0.9500	C42—C43	1.386 (3)
C14—C15	1.395 (2)	C42—H42	0.9500
C14—C21	1.483 (2)	C43—C44	1.376 (3)
C15—C16	1.392 (2)	C43—H43	0.9500
C15—H15	0.9500	C44—C45	1.382 (3)
C16—C31	1.491 (2)	C45—C46	1.376 (3)
C21—C22	1.390 (2)	C45—H45	0.9500
C21—C26	1.391 (2)	C46—H46	0.9500
C22—C23	1.384 (2)		
			
C12—O2—C4	118.13 (14)	F1—C24—C25	118.62 (17)
O1—C1—C2	122.64 (16)	C23—C24—C25	123.09 (16)
O1—C1—C11	120.39 (16)	C24—C25—C26	118.00 (17)
C2—C1—C11	116.97 (15)	C24—C25—H25	121.0
C3—C2—C1	122.68 (18)	C26—C25—H25	121.0
C3—C2—H2	118.7	C25—C26—C21	120.92 (17)
C1—C2—H2	118.7	C25—C26—H26	119.5
C2—C3—C41	126.11 (19)	C21—C26—H26	119.5
C2—C3—H3	116.9	C32—C31—C36	118.52 (16)
C41—C3—H3	116.9	C32—C31—C16	119.79 (15)
O2—C4—H4A	109.5	C36—C31—C16	121.69 (16)
O2—C4—H4B	109.5	C31—C32—C33	121.10 (18)
H4A—C4—H4B	109.5	C31—C32—H32	119.5
O2—C4—H4C	109.5	C33—C32—H32	119.5
H4A—C4—H4C	109.5	C34—C33—C32	118.06 (19)
H4B—C4—H4C	109.5	C34—C33—H33	121.0
C16—C11—C12	119.02 (15)	C32—C33—H33	121.0
C16—C11—C1	122.48 (15)	F2—C34—C33	118.4 (2)
C12—C11—C1	118.42 (15)	F2—C34—C35	118.7 (2)
O2—C12—C13	123.84 (15)	C33—C34—C35	122.91 (18)
O2—C12—C11	114.96 (15)	C34—C35—C36	118.53 (19)
C13—C12—C11	121.13 (15)	C34—C35—H35	120.7
C12—C13—C14	119.82 (15)	C36—C35—H35	120.7
C12—C13—H13	120.1	C35—C36—C31	120.82 (19)
C14—C13—H13	120.1	C35—C36—H36	119.6
C13—C14—C15	119.27 (15)	C31—C36—H36	119.6
C13—C14—C21	120.73 (15)	C46—C41—C42	117.83 (19)
C15—C14—C21	119.95 (15)	C46—C41—C3	122.37 (17)
C16—C15—C14	121.53 (16)	C42—C41—C3	119.79 (19)
C16—C15—H15	119.2	C43—C42—C41	121.1 (2)
C14—C15—H15	119.2	C43—C42—H42	119.4
C15—C16—C11	119.11 (15)	C41—C42—H42	119.4
C15—C16—C31	118.76 (15)	C44—C43—C42	119.18 (19)
C11—C16—C31	122.13 (15)	C44—C43—H43	120.4
C22—C21—C26	118.73 (16)	C42—C43—H43	120.4
C22—C21—C14	120.05 (15)	C43—C44—C45	121.1 (2)
C26—C21—C14	121.18 (15)	C43—C44—Br1	119.81 (15)
C23—C22—C21	120.91 (17)	C45—C44—Br1	119.05 (17)
C23—C22—H22	119.5	C46—C45—C44	119.2 (2)
C21—C22—H22	119.5	C46—C45—H45	120.4
C24—C23—C22	118.35 (17)	C44—C45—H45	120.4
C24—C23—H23	120.8	C45—C46—C41	121.47 (19)
C22—C23—H23	120.8	C45—C46—H46	119.3
F1—C24—C23	118.30 (17)	C41—C46—H46	119.3
			
O1—C1—C2—C3	−5.0 (3)	C22—C23—C24—C25	0.1 (3)
C11—C1—C2—C3	175.70 (17)	F1—C24—C25—C26	−179.59 (16)
C1—C2—C3—C41	179.87 (17)	C23—C24—C25—C26	0.6 (3)
O1—C1—C11—C16	−53.0 (2)	C24—C25—C26—C21	−0.8 (3)
C2—C1—C11—C16	126.29 (18)	C22—C21—C26—C25	0.4 (3)
O1—C1—C11—C12	123.62 (19)	C14—C21—C26—C25	178.02 (16)
C2—C1—C11—C12	−57.1 (2)	C15—C16—C31—C32	−42.3 (2)
C4—O2—C12—C13	−12.6 (3)	C11—C16—C31—C32	137.48 (18)
C4—O2—C12—C11	170.62 (15)	C15—C16—C31—C36	137.16 (18)
C16—C11—C12—O2	−179.64 (15)	C11—C16—C31—C36	−43.1 (2)
C1—C11—C12—O2	3.6 (2)	C36—C31—C32—C33	1.8 (3)
C16—C11—C12—C13	3.4 (3)	C16—C31—C32—C33	−178.73 (16)
C1—C11—C12—C13	−173.33 (16)	C31—C32—C33—C34	0.3 (3)
O2—C12—C13—C14	−178.88 (16)	C32—C33—C34—F2	179.10 (17)
C11—C12—C13—C14	−2.2 (3)	C32—C33—C34—C35	−1.9 (3)
C12—C13—C14—C15	−1.0 (3)	F2—C34—C35—C36	−179.76 (18)
C12—C13—C14—C21	176.46 (16)	C33—C34—C35—C36	1.2 (3)
C13—C14—C15—C16	3.0 (3)	C34—C35—C36—C31	1.0 (3)
C21—C14—C15—C16	−174.42 (16)	C32—C31—C36—C35	−2.5 (3)
C14—C15—C16—C11	−1.8 (3)	C16—C31—C36—C35	178.06 (17)
C14—C15—C16—C31	177.93 (16)	C2—C3—C41—C46	4.2 (3)
C12—C11—C16—C15	−1.4 (2)	C2—C3—C41—C42	−176.57 (19)
C1—C11—C16—C15	175.23 (16)	C46—C41—C42—C43	−1.4 (3)
C12—C11—C16—C31	178.87 (15)	C3—C41—C42—C43	179.34 (19)
C1—C11—C16—C31	−4.5 (3)	C41—C42—C43—C44	−0.1 (3)
C13—C14—C21—C22	−45.3 (2)	C42—C43—C44—C45	1.5 (3)
C15—C14—C21—C22	132.09 (18)	C42—C43—C44—Br1	179.66 (16)
C13—C14—C21—C26	137.12 (18)	C43—C44—C45—C46	−1.2 (3)
C15—C14—C21—C26	−45.5 (2)	Br1—C44—C45—C46	−179.46 (17)
C26—C21—C22—C23	0.3 (3)	C44—C45—C46—C41	−0.3 (3)
C14—C21—C22—C23	−177.33 (16)	C42—C41—C46—C45	1.6 (3)
C21—C22—C23—C24	−0.6 (3)	C3—C41—C46—C45	−179.13 (19)
C22—C23—C24—F1	−179.72 (16)		

### Hydrogen-bond geometry (Å, º)

**Table:**

*D*—H···*A*	*D*—H	H···*A*	*D*···*A*	*D*—H···*A*
C4—H4*B*···F1^i^	0.98	2.51	3.428 (2)	156
C25—H25···O1^ii^	0.95	2.43	3.268 (2)	147

Symmetry codes: (i) *x*+1, *y*+1, *z*; (ii) *x*, *y*−1, *z*.
